# Efficacy and safety of an innovatively modified cutting seton technique for the treatment of high anal fistula: A protocol for a randomized controlled trial

**DOI:** 10.1097/MD.0000000000024442

**Published:** 2021-02-05

**Authors:** Jie Jiang, Yang Zhang, Xufeng Ding, Naijin Zhang, Lijiang Ji

**Affiliations:** a Changshu Hospital Affiliated to Nanjing University of Chinese Medicine, Changshu, Jiangsu province; b Department of Anorectal Surgery, Nanjing Hospital of Chinese Medicine Affiliated to Nanjing University of Chinese Medicine, Nanjing; c Tianjin University of Traditional Chinese Medicine, Tianjin, China.

**Keywords:** cutting, high anal fistula, incise, operation, seton

## Abstract

**Background:**

Anal fistula is a common anorectal disease. So far, operation is still the optimal method to cure anal fistula. High anal fistula (HAF) is an even more clinically difficult disease to treat. Evidence suggested that seton placement can be a definitive treatment for HAF. However, tightening the seton brings great pain to patients, which affects the clinical application of the therapy. Also, this may lead to difficulty in controlling anal fluids and gas because of the larger scar left and the local defect in the anal after the operation. We propose an innovative seton technique for the treatment of HAF, after long term attempts, the operation of the modified seton cutting technique. The aim of our present study is to compare the difference of anal function, healing time, pain severity, recurrence, and complications between the procedure of the modified seton cutting technique and the conventional cutting seton operation against HAF with a randomized, controlled, prospective study.

**Methods:**

204 participants in this trial will be randomly divided into treatment group (procedure of the modified seton cutting technique) and control group (cutting seton technique) in a 1:1 ratio. The outcomes of continence state, pain severity after tightening, complete healing of fistula, duration to healing, operation time, recurrence rates, and postoperative complications will be recorded at 1, 2, 3, 4 weeks, then every month in the outpatient clinic. Data will be analyzed by SPSS version 22.

**Conclusions:**

The findings of the study will help to explore the efficacy and safety of the procedure of the modified seton cutting technique against AF.

**Trial registration number:**

DOI 10.17605/OSF.IO/V6G2S

## 1 Introduction

Anal fistula is a common anorectal disease, with an estimated prevalence of 0.2%.^[1]^ It is often followed with an infection that does not easily heal with medications alone. So far, operation is still the optimal method to cure anal fistula. High anal fistula (HAF) is 1 of the intractable diseases in colorectal surgery, and it is difficult to achieve both goals of eradicating anorectal sepsis and preserving anal function. In recent years, more and more sphincter-sparing techniques have been applied to the treatment of HAF, including seton,^[2,3]^ endoanal advancement flap,^[4]^ ligation of intersphincteric fistula tract,^[5,6]^ and video-assisted anal fistula treatment.^[7]^ Nevertheless, those techniques may result in high recurrence rates.^[8,9]^

Evidence suggested that cutting seton technique can be a definitive treatment for HAF.^[10,11]^ The mechanism by which a drainage seton acts is still not clear, but by chronic strangulation, drainage, and stimulation, the technique can preserve the anal function as much as possible. However, the traditional cutting seton technique often needs 2 or even several times to tighten the thread. Because of the abundant nerves in the anal region, tightening the seton brings great pain to patients, which affects the clinical application of the therapy. In addition, it may lead to difficulty in controlling anal fluids and gas because of the larger scar left and the local defect in the anal after the operation.

Therefore, we propose an innovatively modified seton cutting technique for the treatment of HAF after long term attempts. In the procedure, a drainage seton is rerouted around internal anal sphincter to divide it slowly in the first stage, then loosely tighten the seton to promote the fibrosis of the anorectal ring when the granulation tissue grows close to the seton area in the middle stage, and incised the anorectal ring with a scalpel at last. The procedure could significantly reduce the pain when tightening the seton, shorten the healing time, and effectively shrink the scar, and preserve the anal function. The aim of our present study is to compare the difference of anal function, healing time, pain severity, recurrence, and complications between the procedure of the modified seton cutting technique and the conventional seton cutting technique against HAF with a randomized, controlled, prospective study.

## 2 Methods

### 2.1 Study design

This is a single-center, randomized, and controlled clinical trial. It has been registered in open Science Framework (Registration number: DOI 10.17605/OSF.IO/V6G2S), and approved by the Ethics Committee of Changshu Hospital Affiliated to Nanjing University of Chinese Medicine, and it will be carried out in accordance with the Declaration of Helsinki. The study conforms to the Standard Protocol Recommendations for Interventional Trials 2013 Statement,^[12]^ and the results will be reported according to the CONSORT Statement extension for trials.^[13]^

### 2.2 Participants

Participants will be mainly recruited from inpatients of proctology department in Changshu Hospital Affiliated to Nanjing University of Chinese Medicine. Patients are included if they meet the following criteria:

(1) Patients meeting the diagnostic criteria for AF
(2) Patients aged from 18 to 70 years old;
(3) Patients without obvious deformity of anus before the operation;
(4) Patients confirmed to high anal fistula by intra-anal digital examination and probe examination, intracavitary B ultrasound or pelvic MRI;
(5) Written informed consent.

Patients are excluded if they present the following criteria:

(1) Patients who have undergone AF surgery 2 or more times;
(2) AF due to trauma, tuberculosis, Crohn, ulcerative colitis;
(3) Patients with rectal cancer, rectal polyps, and other anorectal diseases.
(4) Women in pregnancy, lactation and menstruation.

### 2.3 Randomization and blinding

**Figure 1:**
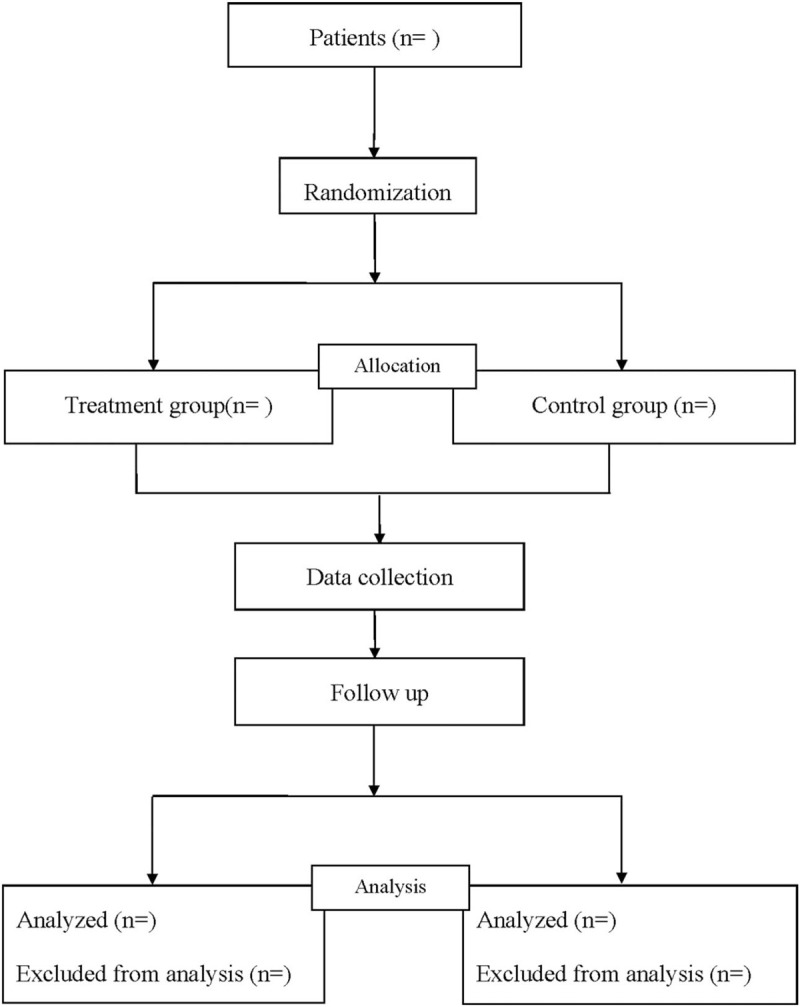
Flow diagram of the study.

The patients will be randomly divided into treatment group and control group in a 1:1 ratio by a computer-generated randomization list (Fig. 1). Blind method is not applicable because of different operations, and an independent professional researcher will analyze the statistic who does not know the identification of the groups.

### 2.4 Interventions

Before the operation, patients will get blood routine test, blood glucose, coagulation, liver and kidney function, infectious disease test series, and electrocardiogram. Intraspinal anesthesia will be performed.

Before the operation, patients will get blood routine test, blood glucose, coagulation, liver and kidney function, infectious disease test series, and electrocardiogram. Intraspinal anesthesia will be performed.

#### 2.4.1 Intervention group

The procedure of the modified seton cutting technique will be performed with the following procedure:

(1) Make sure the position of internal ostium and the path of the fistula and its relationship with the sphincter by digital examination combined with preoperative intracavitary B-ultrasound or MRI;
(2) From the posterior triangular approach of the anal canal, under the guidance of the probe, incise the skin and subcutaneous tissue along the fistula canal 2 cm above the anal margin to expose the posterior rectal space;
(3) Explore the internal ostium and place a rubber seton, and tight the seton after the operation;
(4) Properly trim the edge of the skin to maintain smooth drainage, and completely stop bleeding;
(5) After the operation, when the granulation tissue grows close to the seton area, slightly tighten the seton and then hang it on the wound surface. After the granulation tissue growing close to the seton area again and the area becoming fibrosis, and the second formation of the anorectal ring, directly incise the seton area under local anesthesia.

#### 2.4.2 Control group

Cutting seton technique will be performed with the same procedure with the following procedure:

(1) to (4): The first 4 steps are the same with that in treatment group

(5) After the operation, when the granulation tissue grows close to the seton area, tighten the seton once and every 7 days according to the patient's tolerance to pain and the operator's clinical experience, until the rubber band falls off and the tissue is completely cut.

### 2.5 Outcome variables

The primary outcomes of the study are the continence state after surgery as assessed with Wexner incontinence score,^[14]^ and pain severity after tightening by Visual Analogue Scale. Secondary outcomes include complete healing of fistula, duration to healing, operation time, recurrence rates, and postoperative complications. All possible adverse events will be recorded, such as bleeding, anal stenosis, anal fissure, urinary retention, difficulty in defecation, urgency of stool, pelvic sepsis at any time. Patients will be followed-up at 1, 2, 3, 4 weeks, then every month in the outpatient clinic. At 6 months postoperatively, patients will be clinically assessed for recurrence of AF.

### 2.6 Sample size calculation

According to previous literature^[15]^ that reported fecal incontinence to occur in up to 25.5% of patients with AF after cutting seton technique. Presuming that the rate of fecal incontinence after the operation of the modified seton cutting technique will be 10%, and taking α of 0.05 and β of 0.2, a sample size of 92 in each group will be needed. In order to compensate for loss to follow-up, 204 patients will be ultimately included.

### 2.7 Statistical methods

Data will be analyzed by SPSS version 22(Created by IBM Corporation, New York, America). Statistical testing is 2-sided and *P* < .05 is considered statistically significant. Continuous data are expressed in of mean ± standard deviation, or median (quartile), and categorical variables as number and percentage. Student *t* test will used for normally distributed data and Mann–Whitney *U* test for non-normally distributed data. Fisher test or Chi-squared test will be used to analyze categorical data.

## 3 Discussion

At present, in the surgical treatment of HAF, cutting seton drainage is still the main surgical procedure. By cutting the sphincter slowly with the unique elastic cutting action of the seton, it could induce inflammatory fibrosis, avoid the sphincter retraction and separation, and prevent anal incontinence when the sphincter is momentarily severed.^[11]^ However, there are still some shortcomings according to literature reports.^[16,17]^ First, the pain in the treatment process is severe, because of continuously cutting the fistula and the anal sphincter by the elastic seton. Second, the healing time is longer, which needs about 40 to 50 days. Third, the left scar is large and obvious, which will affect the function of the anal sphincter, leading to incomplete anal incontinence.

The operation of the modified seton cutting is a modified technique of the conventional cutting seton, which could weaken the effect of chronic cutting. The modified technique is supposed to increase the cure rate, reduce the severe pain during the postoperative seton-tightening process, reduce local inflammation and scar, and shorten the healing time. Therefore, we try to conduct a clinical trial to compare the difference of anal function, healing time, pain severity between the procedure of the modified seton cutting technique and the conventional seton placement technique against HAF. However, we need to address several limitations in this protocol. First, the evaluator-blinded-only trial may lead to certain biases. Second, being a single institution study may affect the conclusions.

## Author contributions

**Data collection:** Jie Jiang and Yang Zhang.

**Data curation:** Jie Jiang, Yang Zhang.

**Funding support:** Lijiang Ji.

**Investigation:** Naijin Zhang.

**Literature retrieval:** Yang Zhang and Xufeng Ding.

**Resources:** Yang Zhang, Xufeng Ding.

**Software operating:** Naijin Zhang.

**Software:** Naijin Zhang.

**Supervision:** Naijin Zhang and Lijiang Ji.

**Writing – original draft:** Jie Jiang and Yang Zhang.

**Writing – review & editing:** Jie Jiang and Lijiang Ji.
